# Antimicrobial Potential of Cannabinoids: A Scoping Review of the Past 5 Years

**DOI:** 10.3390/microorganisms13020325

**Published:** 2025-02-02

**Authors:** Maria João Coelho, Maria Duarte Araújo, Márcia Carvalho, Inês Lopes Cardoso, Maria Conceição Manso, Cristina Pina

**Affiliations:** 1 RISE-Health, Faculty of Health Sciences, Fernando Pessoa University, Fernando Pessoa Teaching and Culture Foundation, Rua Carlos da Maia, 296, 4200-150 Porto, Portugal; mcarv@ufp.edu.pt (M.C.); mic@ufp.edu.pt (I.L.C.); cpina@ufp.edu.pt (C.P.); 2 FCS-UFP, Faculdade de Ciências da Saúde (Health Sciences Faculty), Fernando Pessoa University, Rua Carlos da Maia, 296, 4200-150 Porto, Portugal; 38405@ufp.edu.pt; 3 LAQV/REQUIMTE, Department of Chemical Sciences, Faculty of Pharmacy, University of Porto, Rua de Jorge Viterbo Ferreira, 228, 4050-313 Porto, Portugal

**Keywords:** cannabinoids, cannabis, antimicrobial potential, antibacterial, antiviral, antifungal

## Abstract

In the scenario of fighting bacterial resistance to antibiotics, natural products have been extensively investigated for their potential antibacterial activities. Among these, cannabinoids—bioactive compounds derived from cannabis—have garnered attention for their diverse biological activities, including anxiolytic, anti-inflammatory, analgesic, antioxidant, and neuroprotective properties. Emerging evidence suggests that cannabinoids may also possess significant antimicrobial properties, with potential applications in enhancing the efficacy of conventional antimicrobial agents. Therefore, this review examines evidence from the past five years on the antimicrobial properties of cannabinoids, focusing on underlying mechanisms such as microbial membrane disruption, immune response modulation, and interference with microbial virulence factors. In addition, their synergistic potential, when used alongside standard therapies, underscores their promise as a novel strategy to address drug resistance, although further research and clinical trials are needed to validate their therapeutic use. Overall, cannabinoids offer a promising avenue for the development of innovative treatments to combat drug-resistant infections and reduce the reliance on traditional antimicrobial agents.

## 1. Introduction

Antibiotics have long been the treatment strategy for microbial infections. However, the uncontrolled and excessive use of these compounds has led to the development of microorganisms that are resistant to the action of antimicrobial agents, and this has become a problem and a worrying challenge on a global scale. Therefore, one of the priorities of the scientific community has been the search for innovative treatments with novel agents and the exploration of alternative strategies capable of controlling and eradicating these microorganisms and preventing disease, thus promoting global health [1]. 

These new strategies include the investigation of new drug delivery systems and alternative biomolecules with potential antimicrobial activity. 

Regarding new drug delivery systems, several studies have reported the use of a variety of nanomaterials, such as copper, silver, gold, titanium, zinc, and others, in the management of infections. Metallic nanostructures, particularly gold and silver nanoparticles, are widely used in the design of targeted drug delivery systems due to their unique properties, such as stability against corrosion and oxidation, sizes ranging from 1 to 100 nm, good biocompatibility, versatility in surface functionalization, the ability to conjugate drugs through ionic, covalent or physical interactions, and shape-controlled synthesis [2]. These systems enhance the stability, absorption, and circulation of phytocompounds, protecting them from early metabolism and adverse effects. Modified nanocarriers improve the solubility, permeability, and sustained delivery of compounds to targeted diseased sites, thereby improving bioavailability [3]. The work of Alhadrami et al. [4] tested flavonoid-coated gold nanoparticles against Gram-negative bacteria. Also promising are silver nanoparticles, which have shown great potential as nano-fungicides [5], nano-viricides [6], and nano-bactericides [7]. Greatti et al. [8] were successful in using poly-ε-caprolactone nanoparticles with 4-nerolidylcatechol to control the growth of *Microsporum canis*. Preethi and Bellare [9] used another bioflavonoid (quercetin) complexed in magnesium-doped calcium silicates to control Gram-negative bacterial bone infections. These researchers observed a marked reduction in bacterial adhesion and proliferation. Another new vehicle for conventional antibiotic delivery was presented by Lin et al. [10]. In this work, aerosolized hypertonic saline was used to enhance the antibiotic susceptibility of multidrug-resistant *Acinetobacter baumannii*.

In addition to the search and development of new drug delivery systems, the scientific community has also focused on the study of new biomolecules for the treatment of infections and the development of eco-friendly approaches, such as the use of biological extracts and biomolecules to produce antimicrobial agents. These strategies focus on targeting pathogens while reducing the impact on non-target organisms [11]. For example, Han et al. [12] demonstrated the efficacy of grapefruit seed extracts as an antibacterial agent. 

Several studies have also highlighted the importance of sustainable strategies in the synthesis of nanoparticles. As an example, Lakkim et al. [13] developed a new green method for the synthesis of silver nanoparticles and observed an improvement in the performance against antibiotic-resistant bacteria. These new approaches have been combined with the use of *Cannabis sativa* [14]. Singh et al. [15] reported the use of *Cannabis sativa* for the green synthesis of gold and silver nanoparticles and demonstrated their activity in biofilm inhibition, while Chouhan & Guleria [16] characterized the antibacterial and anti-yeast activities of silver nanoparticles prepared with *Cannabis sativa* leaf extracts.

Another recent approach is the use of molecules targeting DNA gyrase and topoisomerase IV to control microbial infections. This was the case of Saleh et al. [17], who tested the efficacy of DNA gyrase inhibitors, diphenylphosphonates, against fluoroquinolone-resistant pathogens. Moreover, monoclonal antibody-based treatments are being investigated for cases of severe sepsis. The advantage of this approach is the specificity of the method since these antibodies target the microorganism or its components and can directly suppress the synthesis of inflammatory agents [18,19,20].

**Figure 1:**
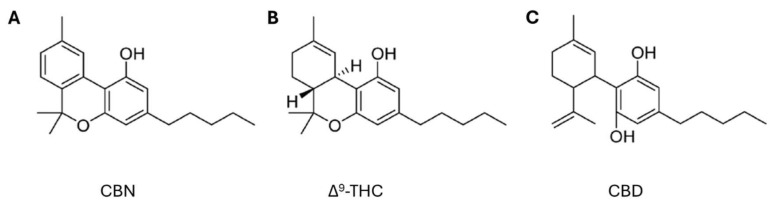
Chemical structures of phytocannabinoids (**A**) cannabinol (CBN), (**B**) delta-9-tetrahydrocannabinol (Δ^9^-THC), and (**C**) cannabidiol (CBD) (adapted from Bow and Rimoldi [26]).

Among these novel strategies, *Cannabis* sp. has received significant attention from the scientific community for its potential applications across diverse therapeutic areas, including the treatment of inflammation, pain management, cancer therapy, neuroprotection, and others [21,22,23]. Cannabis is a herbaceous plant belonging to the *Cannabinaceae* family that has been used for centuries for textile, food, medicinal, and recreational purposes [24]. This plant contains several chemically active compounds, such as cannabinoids, terpenes, alkaloids, and flavonoids. Cannabinoids can be classified into three types: endogenous, known as endocannabinoids, which occur naturally in mammals, the best known being anandamide (AEA) and 2-arachidonoylglycerol (2-AG); phytocannabinoids (Figure 1), which are naturally found in the cannabis plant, including cannabidiol (CBD), delta-9-tetrahydrocannabinol (Δ^9^-THC), cannabigerol (CBG), cannabinol (CBN), and cannabichromene (CBC); and synthetic cannabinoids, produced in the laboratory. All these cannabinoids can bind to cannabinoid receptors type 1 (CB_1_) and type 2 (CB_2_), which are located in the plasma membranes of nerve cells and the immune system, respectively [25].

Over time, phytocannabinoids have proven their antibacterial and anti-inflammatory activity and have potential use in these new therapies [27]. In some cases, researchers have also evaluated the synergistic effect that might exist between cannabinoids and conventional antibiotics, with the aim of antagonizing bacterial resistance mechanisms [24].

This scoping review aims to provide a concise overview of the evidence from the last 5 years (2020–2024) on the therapeutic potential of cannabinoids against different classes of microorganisms (bacteria, viruses, parasites, and fungi), which may pave the way for the development of novel therapies. 

## 2. Methods

### 2.1. Search Strategy

This scoping review was performed according to the Preferred Reporting Items for Systematic Reviews and Meta-Analyses (PRISMA) guidelines [28]. The literature search was conducted on 13 November 2024, using PubMed, Scopus, and ScienceDirect databases, and supplemented by manually searching the reference lists of included studies. The search included all original research papers published from 1 January 2020, to 31 October 2024. PubMed was searched using the following search strategy: (“cannab*” AND (“antimicrobial*” OR “antibacterial*” OR “antiviral*” OR “antifungal*” OR “Antibiofilm”)) NOT “review” [Publication Type]) in the title or abstract. This strategy was then adapted to the syntax and subject headings of the other databases. The detailed search strategy for each database, designed to maximize the retrieval of relevant articles, is presented in Table S1 in the Supplementary Material section.

### 2.2. Inclusion and Exclusion Criteria

Studies were included according to the following criteria: (1) studies written in English; (2) studies evaluating the antimicrobial activities of cannabinoid compounds; (3) studies performed *in vitro* and/or *in vivo*. 

Studies were excluded if they met the following criteria: (1) review articles or conference abstracts; (2) studies not relevant to the review topic; (3) studies evaluating the antimicrobial effects of cannabis extracts, oils, flowers, or pollen; (4) studies not related to human infections; and (5) studies using models other than *in vivo* and *in vitro* models.

### 2.3. Overview of Included Studies

**Figure 2:**
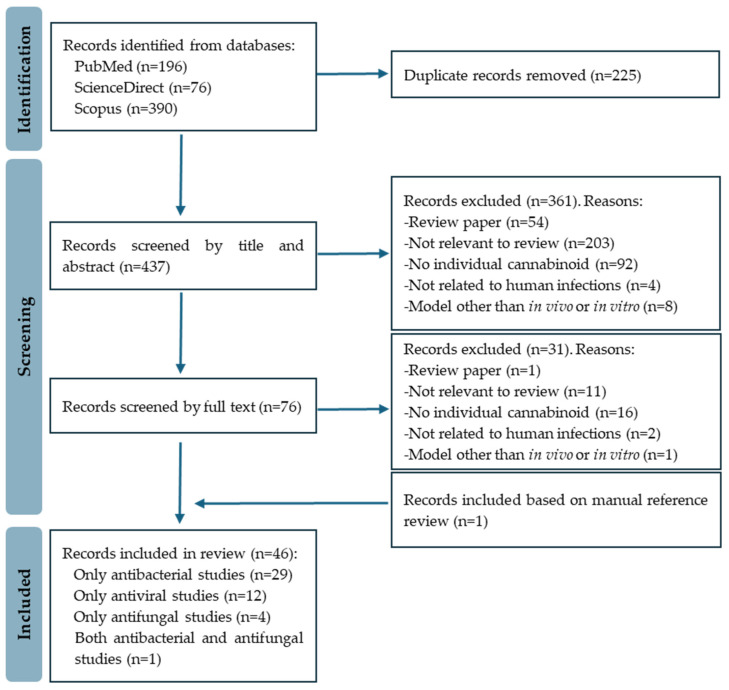
PRISMA flow diagram of study selection.

The initial search yielded a substantial pool of 662 records. The EndNote 20 software was employed to eliminate duplicates, resulting in a refined set of 437 research articles for further review. An initial meticulous screening based on titles and abstracts resulted in the exclusion of 361 records. The full text of the remaining 76 papers was then reviewed, with 46 studies ultimately meeting the eligibility criteria considered for this review. The included studies covered antibacterial (30 papers), antiviral (12 papers), and antifungal (5 papers) activities. Figure 2 depicts the PRISMA flow diagram of the study selection process.

### 2.4. Data Extraction

The following information was extracted from the selected articles: publication details (first author’s last name and publication year), study type, objectives, and key findings. The information is summarized in Table 1, Table 2, and Table 3.

A scoping review provides an overview of the existing evidence, regardless of its quality. Therefore, critical appraisal, also referred to as the assessment of risk of bias or methodological quality assessment, was not conducted for the studies included in this review [29].

## 3. Results

### 3.1. Antibacterial Activity of Cannabinoids

The search strategy of this scoping review led to the selection of 30 publications that studied the antibacterial activity of cannabinoids. Moreover, some studies also discussed the possible mechanisms involved in this activity against bacteria. Table 1 shows the list of the selected papers together with the classification of the type of study, main goals, and results.

**Table 1:** Characteristics of the included studies and the main results of the antibacterial activity of cannabinoids. (Abbreviations: CBD: cannabidiol, CBC: cannabichromene, CBG: cannabigerol, CBN: cannabinol, CBCA: cannabichromenic acid, CBDA: cannabidiolic acid, CBDV: cannabidivarin, CBGA: cannabigerolic acid, EPS: extracellular polysaccharide, H_2_CBD: dihydrocannabidiol, LPS: lipopolysaccharide, MIC: minimum inhibitory concentration, THCBD: tetrahydrocannabidiol, VRE: vancomycin-resistant *E. faecium*, MRSA: methicillin-resistant *S. aureus*, MSSA: methicillin-sensitive *S. aureus*, VISA: vancomycin-intermediate resistant *S. aureus*).

Author (Year)	Type of Study	Aims	Main Results
Abichabki et al. [30]	*In vitro* study	To evaluate the antibacterial activity of CBD against a wide diversity of bacteria and of the combination CBD + polymyxin B (PB) against Gram-negative bacteria, including PB-resistant Gram-negative bacilli.	- Antibacterial activity of CBD against Gram-positive bacteria and *Mycobacterium tuberculosis* was observed, but not against Gram-negative bacteria, probably due to the presence of LPS molecules and outer membrane proteins resulting in impermeability of CBD. - CBD MIC ranged from 2 to 4 μg/mL against Gram-positive bacteria, including VRE, MRSA, and VISA. - For most tested Gram-negative bacteria, including multidrug-resistant and extensively drug-resistant clones (e.g., *Klebsiella pneumoniae, Escherichia coli, Acinetobacter baumannii*), results showed that CBD concentrations lower than 4 μg/mL were sufficient for antibacterial activity in the combination CBD + PB. - PB promotes the destabilization of LPS, leading to the disruption of the Gram-negative bacterial cell envelope and allowing the activity of CBD. - CBD + PB exhibited a synergistic effect, as opposed to when they were used individually at the same concentration.
Aqawi et al. [31]	*In vitro* study	To evaluate the anti-quorum sensing (anti-QS) and anti-biofilm formation potential of CBG on Gram-negative *Vibrio harveyi*.	- CBG showed strong anti-QS and anti-biofilm properties against *V. harveyi* with no detectable MIC and significantly reduced bacterial motility, which plays a key role in biofilm formation. - At sub-MIC concentrations, CBG reduced the amount of bacteria in the biofilms and modified their structure. - The molecular mechanisms used by CBG in the anti-QS activity involve interference with the transmission of the autoinducer signals. - CBG did not affect the growth of *V. harveyi*, but rather interfered with the quorum sensing system.
Aqawi et al. [32]	*In vitro* study	To study the antibacterial activity of CBG against *Streptococcus mutans.*	- CBG exhibited a bacteriostatic effect at a concentration of 2.5 µg/mL, which is affected by the initial bacterial cell density, and a bactericidal effect at higher concentrations of 5–10 µg/mL. - CBG caused intracellular accumulation of mesosome-like structures and increased membrane permeability by causing membrane hyperpolarization and affecting ion channels, leading to changes in cell membrane properties. - CBG inhibited cell division and prevented the drop in pH caused by *S. mutans,* preventing its cariogenic property.
Aqawi et al. [33]	*In vitro* study	To evaluate the potential use of CBG against *S. mutans* biofilms as a means to combat dental plaque.	- CBG increased ROS levels, which might cause oxidative stress in the bacteria. - CBG inhibited the production of extracellular polysaccharides (EPSs); since EPSs prevent the penetration of many antibiotics, the effect of CBG is essential for enhancing the effectiveness of other antibacterial compounds. - CBG reduced the expression of biofilm-regulating genes and inhibited quorum sensing.
Avraham et al. [34]	*In vitro* study	To study the anti-biofilm activity of CBD combined with triclosan against *Streptococcus mutans.*	- The combined treatment of triclosan and CBD had stronger antibacterial and anti-biofilm activities than each compound alone. - Both triclosan and CBD induced membrane hyperpolarization, affecting the viability of the bacteria and their ability to adhere to the surface.
Barak et al. [35]	*In vitro* study	To study the antibacterial and anti-biofilm activities of CBD against *Streptococcus mutans*.	- The MIC of CBD to *S. mutans* was 5 μg/mL. - CBD prevented bacteria-mediated reduction in pH values and decreased EPS production. - Despite the absence of bacteria, EPSs were still detected, although at a much lower level, suggesting that the extracellular enzymes released by the bacteria remained active and continued to synthesize EPSs independently, thereby contributing to the formation of the biofilm extracellular matrix. - CBD reduced the viability of *S. mutans* biofilms at 7.5 μg/mL.
Blaskovich et al. [36]	*In vitro, ex vivo,* and *in vivo* studies	To evaluate the antibacterial activity of CBD against Gram-positive and Gram-negative bacteria.	- CBD showed antibacterial effects against antibiotic-sensitive and antibiotic-resistant *Staphylococcus aureus,* with an MIC of around 1–5 µg/mL. - CBD also had an antibacterial effect on other Gram-positive bacteria (e.g., *Streptococcus pneumoniae* and *Clostridioides difficile*), as well as a subset of Gram-negative bacteria, e.g., *Neisseria gonorrhoeae*. - The primary antibacterial action of CBD was destruction of the membrane. - CBD did not lead to resistance after repeated exposure.
Cham et al. [37]	*In vitro* study	To evaluate the antibacterial potential of a semisynthetic phytocannabinoid, tetrahydrocannabidiol (THCBD, 4) against sensitive and resistant strains of *Staphylococcus aureus*.	- CBD showed potent activity against *S. aureus* ATCC-29213 and *E. coli* ATCC-25922, with an MIC of 4 μg/mL. - THCBD, 4 showed antibacterial activity against those bacteria but at a lower MIC against *S. aureus* ATCC-29213 (0.25 μg/mL). - THCBD, 4 showed strong effectiveness against efflux pump-overexpressing strains as well as MRSA. - THCBD, 4 demonstrated additive effects with tetracycline, mupirocin, and penicillin G.
Cohen et al. [38]	*In vitro, ex vivo,* and clinical studies	To evaluate the efficacy of a newly developed natural topical formulation based on CBD for the treatment of acne.	- CBD at 5 and 10 μg/mL reduced *Cutibacterium acnes* growth in a comparable manner to ampicillin. - CBD significantly decreased the secretion of both TNFα and IL-1β. - CBD combined with *Centella asiatica* triterpene extract or silymarin had superior anti-inflammatory activity to either ingredient alone.
Farha et al. [39]	*In vitro* and in *vivo* studies	To study the antibacterial activity of cannabinoids against MRSA.	- CBG, CBD, CBN, CBCA, and THC (Δ^8^ and Δ^9^) showed potent antibacterial activity, with an MIC value of 2 μg/mL. - The same cannabinoids inhibited MRSA’s ability to form biofilms, with CBG exhibiting the most potent antibiofilm activity (0.5 μg/mL (1/4 MIC)). - CBG eradicated preformed MRSA at 4 μg/mL and stationary phase cells persistent to conventional antibiotics such as gentamicin, ciprofloxacin, and vancomycin. - CBG did not lead to resistance against MRSA. - CBG acts by perturbing the plasma membrane of Gram-positive bacteria. - CBG, which was inactive against *E. coli* (>128 μg/mL), was strongly potentiated when combined with a sublethal concentration of polymyxin B (1 μg/mL in the presence of 0.062 μg/mL polymyxin B). - CBG was effective against Gram-negative bacteria whose outer membrane was permeabilized with polymyxin B, acting on the inner membrane.
Galletta et al. [40]	*In vitro* study	To study the ability of the phytocannabinoid CBCA and its related synthetic analogs to successfully inhibit the growth of MRSA and other clinically relevant pathogenic bacteria.	- CBCA demonstrated antibacterial activity against MRSA, methicillin-sensitive *S. aureus* (MSSA), and VRE. - CBCA exhibited superior bactericidal activity, both faster and more potent, compared to vancomycin, the current standard treatment for MRSA infections. - CBCA demonstrated antibacterial activity against both exponential and stationary-phase MRSA. - CBCA caused rapid *Bacillus subtilis* cell lysis, enabling reduced treatment time to help prevent the development of antimicrobial resistance to this compound. - The bactericidal activity of CBCA was due to the impairment of the bacterial lipid membrane, maintaining the peptidoglycan wall intact.
Garzón et al. [41]	*In vitro* study	To evaluate the antimicrobial and antibiofilm properties and the immune modulatory activities of CBD and CBG on oral bacteria and periodontal ligament fibroblasts.	- Both cannabinoids demonstrated activity against *Streptococcus mutans*, but CBG (MIC = 10 μM) showed better results than CBD (MIC = 20 μM). - - CBD and CBG reduced multispecies biofilm metabolic activity, and CBD had an effect on biofilms that had already developed.
Gildea et al. [42]	*In vitro* study	To evaluate the antibacterial potential of CBD against *Salmonella newington* and *Salmonella typhimurium*.	- CBD exhibited antibacterial activity against *S. typhimurium* and *S. newington* by causing membrane integrity disruption. - CBD was effective against *S. typhimurium* biofilm at a concentration of 0.125 mg/mL. - CBD inhibited *S. typhimurium* and *S. newington* at lower MIC concentrations (0.125 mg/mL) compared to ampicillin (0.5 mg/mL). - - *S. typhimurium* and *S. newington* developed resistance against CBD, but the mechanism of resistance was not determined.
Gildea et al. [43]	*In vitro* study	To evaluate the potential synergy between CBD and three broad-spectrum antibiotics (ampicillin, kanamycin, and polymyxin B) for potential CBD-antibiotic co-therapy.	- *S. typhimurium* growth was inhibited at very low concentrations of CBD–antibiotic co-therapy (0.5 mg/mL ampicillin + 1 mg/mL CBD and 0.5 mg/mL polymyxin B + 1 mg/mL CBD). - - Co-treatment with CBD and kanamycin showed no significant difference in the growth of S. *typhimurium* compared to kanamycin treatment alone.
Hongsing et al. [44]	*In vitro* and in *vivo* studies	To evaluate the antimicrobial efficacy of CBD against clinical isolates of multi-drug resistant *Enterococcus faecalis* bacterial infections *in vitro* and *in vivo*.	- CBD exhibited antibacterial activity against *E. faecalis* biofilm at a lower MIC (2 mg/mL) compared to conventional antibiotics (vancomycin, levofloxacin, and daptomycin). - Mice treated with CBD (100 mg/kg) showed a significant decrease in *E. faecalis* bacterial load in internal organs and improved survival.
Hussein et al. [45]	*In vitro* study	To study the mechanisms of the antibacterial killing synergy of the combination of polymyxin B with CBD against *A. baumannii* ATCC 19606. The antibacterial synergy of the combination against a panel of Gram-negative pathogens (*Acinetobacter baumannii*, *Klebsiella pneumoniae,* and *Pseudomonas aeruginosa*) was also explored using checkerboard and static time–kill assays.	- -Polymyxin B–CBD combination showed synergistic antibacterial activity. - - The metabolomic study demonstrated that polymyxin B monotherapy and in combination significantly perturbed the complex interrelated metabolic pathways involved in the biogenesis of the bacterial cell envelope (amino sugar and nucleotide sugar metabolism, peptidoglycan, and LPS biosynthesis) and nucleotide (purine and pyrimidine metabolism) and peptide metabolism.
Jackson et al. [46]	*In vitro* study	To test the antibiotic potential of CBD, CBC, CBG, and their acidic counterparts (CBDA, CBGA, and CBCA) against Gram-positive bacteria and explore the additive or synergistic effects with silver nitrate or silver nanoparticles.	- All six cannabinoids had strong antibiotic effects against MRSA, with MICs of 2 mg/L for CBG, CBD, and CBCA; 4 mg/L for CBGA; and 8 mg/L for CBC and CBDA. - CBC, CBG, and CBGA showed full or partial synergy with silver nitrate; CBC, CBDA, and CBGA were fully synergistic with silver nanoparticles against MRSA.
Kesavan Pillai et al. [47]	*In vitro* study	To evaluate the antimicrobial activity of solubilized CBD against Gram-negative and Gram-positive bacterial strains.	- - CBD solubilized in an organic medium showed no activity against Gram-negative bacterial strains (*E. coli*, *P. aeruginosa*) but high activity against Gram-positive bacterial strains (*S. aureus*, *S. epidermidis*, and *C. acnes*).
Luz-Veiga et al. [48]	*In vitro* study	To study CBD and CBG interaction and their potential antimicrobial activity against selected microorganisms (human-skin-specific microorganisms commonly associated with inflammatory skin conditions).	- Both cannabinoids showed activity against planktonic bacteria and biofilms by removing mature biofilms at concentrations below the determined MIC. - CBD and CBG exhibited antimicrobial activity against *Pseudomonas aeruginosa* and *E. coli* (MIC ranging from 400 to 3180 µM) and demonstrated an ability to inhibit *Staphylococci* adhesion to keratinocytes, with CBG showing greater activity than CBD.
Martinena et al. [49]	*In vitro* study	To investigate the antimicrobial effect of CBD on *Mycobacterium tuberculosis* intracellular infection.	- CBD exhibited antimicrobial activity against *M. smegmatis* (MIC = 100 μM) and *M. tuberculosis* H37Rv (MIC = 25 μM)
Martinenghi et al. [50]	*In vitro* study	To evaluate the antimicrobial effect of CBDA and CBD.	- CBD displayed a substantial inhibitory effect on Gram-positive bacteria, with minimal inhibitory concentrations ranging from 1 to 2 µg/mL. - Time–kill analysis and minimal bactericidal concentration revealed potential bactericidal activity of CBDA and CBD. - Cannabinoids showed a significant antimicrobial effect on the Gram-positive *S. aureus* and *Staphylococcus epidermidis*, but no activity was noticed on Gram-negative *E. coli* and *Pseudomonas aeruginosa*.
Poulsen et al. [51]	*In vitro* study	To investigate the antibacterial activities of CBD, CBN, and THC against MRSA strains.	- CBD, CBN, and THC showed antibiotic activity against MRSA. - Subjecting MRSA to nonlethal levels of methicillin resulted in increased production of penicillin-binding protein 2 (PBP2). - Reduced levels of proteins involved in energy and PBP2 production were observed when using cannabinoids in combination with methicillin.
Russo et al. [52]	*In vitro* study	To compare the antibacterial activities of CBD and CBDV against *E. coli* and *S. aureus*.	- No evident differences in antimicrobial activity were observed between the two cannabinoids, except with respect to *S. aureus*, which showed greater susceptibility to CBD than to CBDV after 72 h of exposure.
Shi et al. [53]	*In vitro* study	To determine the anti-inflammatory activity of dihydrocannabidiol, (H_2_CBD) and its antibacterial properties against *E. faecalis* and *B. cereus*.	- CBD and H_2_CBD exhibited almost identical performances in all the assayed anti-inflammatory properties, but their anti-inflammatory efficiencies positively correlated with their antioxidative activities. - CBD and H_2_CBD also showed strong and very similar antibacterial activities, comparable to tetracycline in the same dose and strength. - All combinations of H_2_CBD with other cannabinoids or antibiotics demonstrated no antagonism against the bacteria but showed synergistic or additive effects in some cases.
Stahl & Vasudevan [54]	*In vitro* study	To compare the efficacy of oral care products and cannabinoids (CBD, CBC, CBN, CBG, and CBGA) in reducing the bacterial content of dental plaques.	- All tested cannabinoids were more effective at reducing the bacterial colony count in dental plaques compared to well-established synthetic oral care products such as Oral B and Colgate.
Valh et al. [55]	*In vitro* study	To determine the antioxidant and antibacterial activities of microencapsulated CBD against *E. coli* and *S. aureus.*	- CBD–liposome-functionalized tampons have both antioxidant and antimicrobial properties. - Antimicrobial properties were more pronounced against Gram-positive bacteria. - The prepared CBD–liposome-functionalized tampon showed higher biodegradability compared to references.
Vasudevan & Stahl [56]	*In vitro* study	To evaluate CBD and CBG-infused mouthwash products against aerobic bacterial content from dental plaque samples.	- CBD and CBG-infused mouthwash products (containing < 1% cannabinoid by weight) showed similar bactericidal efficacy as that of chlorhexidine 0.2%. - Both chlorhexidine 0.2% and cannabinoid-infused mouthwash products were effective against all the samples tested. - The ranges of zones of inhibition were 8-25 mm for CBD and CBG-infused mouthwash and 12-25 mm for chlorhexidine 0.2%. - - No significant difference was observed between CBD and CBG-infused mouthwash.
Wassmann et al. [57]	*In vitro* study	To characterize CBD as a helper compound against resistant bacteria.	- CBD potentiates the effect of bacitracin against Gram-positive bacteria (*Staphylococcus* species, *Listeria monocytogenes*, and *Enterococcus faecalis*) but appears ineffective against Gram-negative bacteria. - Morphological changes in *S. aureus* as a result of the combination of CBD and bacitracin included several septa formation during cell division along with membrane irregularities.
Wu et al. [58]	*In vitro* study	To determine the antibacterial, bactericidal, and antioxidant activities of 8,9-dihydrocannabidiol against *S. aureus* and *E. coli*.	- The phenolic hydroxyl moiety is an essential group required for CBD analogs’ antibacterial and antioxidant activities. - H_2_CBD demonstrated much stronger antibacterial activity than the assayed popular antibiotics. - H_2_CBD demonstrated lower toxicity to human skin fibroblasts at concentrations up to 64-fold higher than its MIC value (1.25 μg/mL) against *S. aureus.* - H_2_CBD demonstrated extremely similar performance to CBD
Zhang et al. [59]	*In vitro* study	To study the antibacterial activities of a series of novel CBD derivatives against MRSA.	- Gram-positive bacteria (*S. aureus*, *S. epidermidis,* and MRSA) were effectively inhibited by CBD derivatives. - Derivative 21f showed augmented antibacterial activity against MRSA, with a minimum inhibitory concentration of 4 μM without cytotoxic effect in microglia BV2 cells. - 21f inhibited the formation of biofilms, induced excess reactive oxygen species formation, and reduced bacterial metabolism; these collectively led to the acceleration of bacterial death.

### 3.2. Antiviral Activity of Cannabinoids

The search strategy of this scoping review led to the selection of 12 publications that investigated the antiviral activity of cannabinoids. In addition, some studies also discussed the possible mechanisms involved in this activity against viruses. Table 2 shows the list of the selected papers together with the classification of the type of study, main goals, and results.

**Table 2:** Characteristics of the included studies and the main results of the antiviral activity of cannabinoids. (Abbreviations: CBG: cannabigerol, CBL: cannabicyclol, CBN: cannabinol, CBD: cannabidiol, CBDA: cannabidiolic acid, CBGA: cannabigerolic acid, NSAIDs: non-steroidal anti-inflammatory drugs, THC: tetrahydrocannabinol).

Author (Year)	Type of Study	Aims	Main Results
Classen et al. [60]	*In vitro* study	To test synthetic CBG and CBL for potential antiviral effects against SARS-CoV-2.	- CBG and CBL at concentrations ≤ 20 µM exhibited anti-SARS-CoV-2 activity by reducing viral entry by affecting the virus’ spike protein-mediated membrane fusion.
Marques et al. [61]	*In vitro* study	To evaluate the effect of three derivatives and an analog of CBD on non-infected VERO cell viability and antiviral activities against SARS-CoV-2.	- All cannabinoids showed no cytotoxicity at the maximum concentration of 100 μM, and all of them demonstrated promising *in vitro* anti-SARS-CoV-2 activity.
Marquez et al. [62]	*In vitro* study	To explore the *in vitro* antiviral activity of CBD against ZIKV, as well as expanding to other dissimilar viruses.	- CBD exhibited potent antiviral activity against all the tested viruses in different cell lines, with half maximal effective concentration values ranging from 0.87 to 8.55 mΜ. - CBD affected cellular membranes, thus affecting the multiplication of ZIKV and other viruses.
Nguyen et al. [63]	*In vitro* study	To determine CBD’s potential to inhibit infection of cells by SARS-CoV-2	- CBD potently inhibited viral replication under nontoxic conditions, with a median effective concentration (EC_50_) of ~1 μM. - CBD inhibited the ability of three SARS-CoV-2 variants (α, β, and γ) and the original SARS-CoV-2 strain to infect cells. - Related CBD congeners (THC, CBDA, cannabidivarin, cannabichromene, and CBG) were not capable of inhibiting SARS-CoV-2 infection. - Combining CBD with THC (1:1) significantly suppressed CBD efficacy, consistent with competitive inhibition by THC.
Pawełczyk et al. [64]	*In vitro* study	To explore the potential of the molecular consortia of CBD and NSAIDs (ibuprofen, ketoprofen, and naproxen) as novel antiviral dual-target agents against SARS-CoV-2.	- CBD–Naproxen use led to a significant reduction in SARS-CoV-1 virus entry (with IC_50_ below 1 μg/mL). This effect was concentration-dependent. - No significant inhibitory effects of CBD–Ibuprofen and CBD–Ketoprofen on SARS-CoV-1 virus entry were observed. - CBD–Ibuprofen and CBD–Naproxen molecular consortia significantly inhibited SARS-CoV-2 entry (with IC_50_ below 1 μg/mL). - The weakest inhibitory effect on SARS-CoV-2 entry was found in cells treated with CBD–Ketoprofen. - Some CBD–NSAID molecular consortia have superior antiviral activities against SARS-CoV-1 and SARS-CoV-2 but not against the influenza A virus.
Pitakbut et al. [65]	*In vitro* study	To determine the mechanism of action of THC, CBD, and CBN against SARS-CoV2 infection.	- Only CBD acted as a potent viral main protease inhibitor at an IC_50_ value of 1.86 ± 0.04 µM and exhibited only moderate activity against human angiotensin-converting enzyme 2 at an IC_50_ value of 14.65 ± 0.47 µM. - THC acted as a moderate inhibitor against both viral main protease and human angiotensin-converting enzymes at IC_50_ values of 16.23 ± 1.71 µM and 11.47 ± 3.60 µM, respectively.
Polat et al. [66]	*In vivo* study	To determine the antiviral activity of CBD against SARS-CoV-2 infection in K18-hACE2 transgenic mice.	- While the disease progressed and resulted in death in the control group that was infected by the virus alone, it was observed that the infection slowed down, and the survival rate increased in the virus-infected mice treated with CBD.
Raj et al. [67]	*In vitro* study	To estimate the antiviral activity of cannabinoids (CBD, CBN, CBDA, Δ^9^-THC, Δ^9^-THCA) against SARS-CoV-2.	- Two CBD molecules (Δ^9^-THC (IC_50_= 10.25 μM) and CBD (IC_50_= 7.91 μM)) were more potent antiviral molecules against SARS-CoV-2 compared to the reference drugs lopinavir, chloroquine, and remdesivir (IC_50_ ranges of 8.16-13.15 μM).
Santos et al. [68]	*In vitro* study	To evaluate the combination of CBD and terpenes in reducing SARS-CoV-2 infectivity.	- Formulations containing terpenes and CBD (at a concentration of 1 µg/mL) reduced the infectivity of all tested cell lines (from 17% to 99%).
Tamburello et al. [69]	*In vitro* study	To evaluate the antiviral activity of CBDA against SARS-CoV-2.	- CBDA showed the highest antiviral activity against a panel of SARS-CoV-2 variants. - CBDA methyl ester had a neutralizing effect against all the SARS-CoV-2 variants tested, with greater activity than the parent compound.
Van Breemen et al. [70]	*In vitro* study	To determine the antibacterial activities of cannabinoid acids against SARS-CoV-2.	- Cannabinoid acids were found to be allosteric as well as orthosteric ligands with micromolar affinity for the spike protein. - CBGA and CBDA prevented infection of human epithelial cells by a pseudovirus expressing the SARS-CoV-2 spike protein and prevented entry of live SARS-CoV-2 into cells. - CBGA and CBDA were equally effective against the SARS-CoV-2 alpha variant B.1.1.7 and the beta variant B.1.351.
Zargari et al. [71]	*In vitro* study	To test the antiviral activity of 7α-acetoxyroyleanone, curzerene, incensole, harmaline, and CBD against SARS-CoV-2.	- CBD and 7α-acetoxyroyleanone, compounds with the highest binding energy, demonstrated the most inhibitory potential. - The least inhibitory effects were related to the curzerene and incensole structures due to the lowest binding affinities.

### 3.3. Antifungal Activity of Cannabinoids

The search strategy of this scoping review led to the selection of 5 publications that evaluated the antifungal activity of cannabinoids. Moreover, some studies also discussed the possible mechanisms involved in this activity against fungi. Table 3 shows the list of the selected papers together with the classification of the type of study, the main objectives, and the results.

**Table 3:** Characteristics of the included studies and the main results of the antifungal activity of cannabinoids. (Abbreviations: ADH5: alcohol dehydrogenase 5 (class III), CBD: cannabidiol, EPS: extracellular polysaccharide, DPP3: dipeptidyl peptidase 3, ROS: reactive oxygen species, SOD: superoxide dismutase).

Author (Year)	Type of Study	Aims	Main Results
Bahraminia et al. [72]	*In vitro* study	To determine the antifungal activity of CBD against *Candida albicans*.	- CBD at 20 μg/mL inhibited the growth of *C. albicans*. - A decrease in yeast-to-hyphae transition was observed. - Biofilm formation was also significantly reduced. - The inhibition of *C. albicans* was through an apoptosis/necrotic pathway. - CBD synergistically interacted with amphotericin-B to inhibit *C. albicans* growth.
Feldman et al. [73]	*In vitro* study	To determine the potential anti-biofilm activity of CBD and to investigate its mode of action against *C. albicans*.	- CBD inhibited the formation of *C. albicans* biofilm in a concentration- and time-dependent manner. - CBD induced disorganization of mature biofilm at a concentration range below minimal inhibitory and fungicidal concentrations. - CBD reduced biofilm thickness and EPS production by downregulating the *ADH5* gene responsible for the production of the extracellular matrix, as well as *FKS1* and *BIG1* genes required for the synthesis of -1,6-glucan—the main component of EPSs. - CBD inhibited yeast-to-hyphae transition and hyphal growth that support biofilm development; it stimulated the growth of clustered yeast and the appearance of pseudohyphae forms. - CBD repressed the expression of *C. albicans* virulence-associated genes (lipases, phospholipases, and cell wall proteins). - CBD enhanced the production of ROS, reduced the antioxidant defense gene *SOD* and caused mitochondrial dysfunction, and reduced intracellular ATP levels and subsequent apoptosis of *C. albicans* cells. - CBD inhibited *C. albicans* biofilm formation by upregulating the *DPP3* gene (associated with the biosynthesis of farnesol that inhibits biofilm formation) and by downregulating ergosterol biosynthesis-associated genes (*ERG11* and *ERG20*).
Feldman et al. [74]	*Ex vivo* and in *vivo* studies	To investigate the possibility of incorporating CBD, triclosan, and CBD/triclosan into a sustained-release varnish SRV (SRV-CBD, SRV-triclosan) to increase the pharmaceutical potential against *C. albicans* biofilm.	- SRV-triclosan and SRV-CBD strongly inhibited *C. albicans* biofilm formation and showed a sustained inhibitory effect on the development of hyphae, which is recognized as a key pathogenic mechanism of *C. albicans.* - Antibiofilm activity was enhanced in the presence of SRV-CBD–triclosan.
Kesavan Pillai et al. [47]	*In vitro* study	To evaluate the antimicrobial activity of solubilized CBD against fungal strains (*C. albicans*, *M. furfur*).	- MIC values for CBD-IS (isolate with 99.5% purity) against *C. albicans* and *M. furfur* were 25 times higher than the literature-reported MIC value (12.46 μg/mL).
Ofori et al. [75]	*In vitro* study	To assess the anti-*Candida* properties of newly synthesized abnormal CBD derivatives (AbnCBD)	- AbnCBD derivatives (synthetic cannabinoids without psychotropic activities) induced differential inhibition of *Candida* growth (*C. albicans*, *C. tropicalis,* and *C. parapsilosis* but not *C. auris*). - The tested compound also disrupted *C. albicans* biofilm formation and eradicated mature biofilms.

## 4. Discussion

### 4.1. Antibacterial Activity of Cannabinoids—Recent Findings and Interpretation

The growing resistance of bacteria to antibiotics is one of the greatest challenges facing global public health, making the discovery of new therapies an urgent priority. This need is even more pressing and challenging in the case of Gram-negative bacteria, whose cell wall creates a more efficient permeability barrier to external agents. Antimicrobial resistance is also on the rise among Gram-positive bacteria. Among these, *Staphylococcus aureus* and its most resistant form, methicillin-resistant *Staphylococcus aureus* (MRSA), stand out as the cause of hospital- and community-associated infections worldwide and are also an important factor in morbidity and mortality [76]. In this context, the antibacterial properties of cannabinoids have attracted considerable attention.

Several studies have demonstrated the significant antibacterial activity of cannabinoids (Δ^9^-THC, CBD, CBC, and CBG, and their acid forms CBDA, CBCA, and CBGA), especially against Gram-positive bacteria, including *S. aureus* [52,55,58,59], vancomycin-resistant *Enterococcus faecium* (VRE), MRSA, and vancomycin-intermediate resistant (VISA) *S. aureus* [30,36,39,40,46,50,51], as well as the oral cariogenic *Streptococcus mutans* [32,35,41]. CBD and CBG showed a range of MIC values between 2 and 5 µg/mL against several Gram-positive bacteria [30,35,36,37,39,44,50], and neither of them demonstrated resistance after repeated exposure [36,39]. Against *S. mutans*, CBG demonstrated a bacteriostatic effect at 2.5 µg/mL, which was influenced by the initial bacterial cell density, and a bactericidal effect at higher concentrations of 5–10 µg/mL [32]. Concentrations of 5 and 10 μg/mL of CBD reduced the growth of *Cutibacterium acnes* [38]. Galletta et al. [40] reported that CBCA was especially potent against MRSA, exhibiting faster and more effective bactericidal activity compared to vancomycin, particularly against exponential- and stationary-phase bacteria. The authors also demonstrated that this cannabinoid induced rapid *Bacillus subtilis* cell lysis, allowing for a shorter treatment duration, which helps to minimize the risk of antimicrobial resistance developing against this compound [40]. 

Although cannabinoids have been shown to inhibit the growth of a variety of Gram-positive bacteria, their antimicrobial activity against Gram-negative bacteria is limited, possibly due to the presence of the outer membrane, lipopolysaccharides, and porins, which are critical factors in bacterial resistance to external agents. These bacteria are thus impermeable to macromolecules and allow only limited diffusion of hydrophobic molecules [27,30,47,50].

Nevertheless, Gildea et al. [42] observed that CBD exhibited an antibacterial effect against two strains of Gram-negative bacteria, *Salmonella typhimurium* and *Salmonella Newington*, through the disruption of cell membrane integrity. In addition, CBD was found to have antibacterial activity similar to that of ampicillin, with a minimum inhibitory concentration (MIC) approximately one-fifth that of ampicillin. However, the two strains also developed resistance to CBD after 48 hours, suggesting that the mechanism of resistance of the strains to CBD may be different from the mechanism of resistance to antibiotics. Finally, the authors found that CBD, in combination with ampicillin, was effective against biofilms of *S. typhimurium* [42].

Additionally, other studies have demonstrated the antibacterial efficacy of CBD against Gram-negative bacteria, including *E. coli* ATCC-25922 (with a MIC of 4 μg/mL [37]), *Neisseria gonorrhoeae* [36], *E. coli,* and *Pseudomonas aeruginosa* [48].

Studies by Martinena et al. [49] concluded that CBD has a moderate but selective effect on inhibiting the growth of *Mycobacterium smegmatis* (MIC = 100 μM) and *Mycobacterium. tuberculosis* H37Rv (MIC = 25 μM) [49].

Antimicrobial resistance is a major concern in infectious diseases and is closely related to the formation of bacterial biofilms. A biofilm is a structured community of microorganisms embedded in a self-produced matrix of extracellular polymeric substances (EPS) attached to a surface. The biofilm formation process is complex and involves the clustering of microbial cells within an EPS matrix composed of polysaccharides, proteins, lipids, and nucleic acids, which together create a three-dimensional structure. Biofilms contain multiple microbial species that work synergistically within this matrix, protecting microorganisms from antimicrobial agents and the immune system. Biofilms also harbor antibiotic-resistant and persister cells that can survive and regrow after antibiotic treatment [77]. As these biofilm communities contribute to the virulence of infections and play a key role in bacterial relapses and chronic infections, it is essential to evaluate the ability of cannabinoids to eliminate them.

In the study of Fahra et al. [39], CBG, CBD, CBN, CBCA, and Δ^9^-THC demonstrated an ability to inhibit biofilm formation by MRSA, with CBG being the most potent. In the same study, CBG eliminated preformed MRSA biofilms at 4 μg/mL and was effective against stationary-phase cells that were resistant to conventional antibiotics such as gentamicin, ciprofloxacin, and vancomycin [39]. Aqawi et al. [31] reported the anti-biofilm potential of CBG against *Vibrio harveyi* that exhibited strong anti-quorum sensing activity by disrupting the transmission of autoinducer signals, thereby interfering with bacterial communication without affecting the bacteria’s growth. CBG also reduced bacterial motility (a key factor in biofilm formation), disrupted biofilm structure at sub-MIC concentrations, and reduced the expression of biofilm-regulating genes [31,32]. In recent studies, CBD reduced the viability of *S. mutans* biofilms at 7.5 μg/mL and decreased the metabolic activity of multispecies biofilms [35,41]. It also influenced mature biofilms and was effective against *Salmonella typhimurium* biofilms at 0.125 μg/mL and *Enterococcus faecalis* biofilms at low concentrations (2 μg/mL), with its activity sometimes surpassing those of conventional antibiotics (vancomycin, levofloxacin, and daptomycin) [42,44]. Biofilms exhibit increased resistance to antibacterial treatments due to their structural complexity, which impedes drug penetration, and the low metabolic activity of the sessile bacteria within them. Consequently, eradicating bacteria in biofilms poses a greater challenge compared to targeting planktonic bacteria. Despite these challenges, Barak et al. [35] demonstrated that CBD effectively inhibited both planktonic growth and biofilm formation of *S. mutans,* with the MIC and minimum biofilm inhibitory concentration both being 5 µg/mL. In another study, Aqawi et al. [33] showed that CBG also exhibits this dual antibacterial/ antibiofilm activity against *S. mutans* at concentrations as low as 2.5 µg/mL. CBG, similar to CBD, disrupts biofilm formation both indirectly through its antibacterial properties and directly by targeting metabolic pathways essential for biofilm regulation. Specifically, CBG reduces the expression of key biofilm-regulating genes, inhibits EPS production, disrupts quorum sensing, increases ROS production, and suppresses bacterial metabolic activity [33].

Although the mechanism of antimicrobial action of cannabinoids is not yet fully understood, Wassman et al. [57] suggested that this mechanism involves damage to the bacterial cell membrane. Blaskovich et al. [36] also showed that bactericidal concentrations of CBD against *S. aureus* inhibit the synthesis of proteins, DNA, RNA, and peptidoglycan.

CBD and CBG appear to disrupt the plasma membrane of Gram-positive bacteria by distinct mechanisms, as reported in several studies [36,39,43]. These compounds exhibit a bacteriostatic effect by inducing membrane hyperpolarization and disrupting ion channel function, which leads to the intracellular accumulation of mesosome-like structures [32,34]. Additionally, they can prevent bacteria-mediated pH reduction [32,35] and suppress the production of EPS. This suppression enhances the penetration of antibiotics, thereby improving the effectiveness of other antibacterial agents [33,35].

Galletta et al. [40] showed that the bactericidal effect of CBCA resulted from damage to the bacterial lipid membrane while maintaining the integrity of the peptidoglycan wall. This suggests an alternative mechanism of action that reduces the likelihood of existing or cross-antimicrobial resistance [40].

Cham et al. [37] demonstrated that THCBD had strong effectiveness against efflux pump-overexpressing strains. Efflux pumps play a key role in antibiotic resistance by expelling various antibiotics, either individually or cooperatively. Additionally, efflux pumps contribute to bacterial infection spread through biofilm formation by influencing physical–chemical interactions, mobility, gene regulation, and quorum sensing. They also release EPSs and harmful metabolites [78]. 

As research into the antibacterial properties of cannabinoids advances, exploring their potential synergy with other therapeutic agents becomes increasingly important. Co-therapy has long been a strategy for treating resistant bacterial infections, highlighting the need to investigate interactions between cannabinoids, particularly CBD, and broad-spectrum antibiotics in such treatments.

Polymyxin B is an antibiotic used in clinical settings to treat severe healthcare-associated infections caused by multidrug-resistant and extensively drug-resistant Gram-negative bacilli, especially those caused by *P. aeruginosa, E. coli*, *Klebsiella pneumoniae, A. baumannii,* and carbapenem-resistant Enterobacteriaceae [79]. Polymyxin B exerts antibacterial activity through electrostatic interaction between its positive charge and the negative phosphate groups on lipid A in the outer membrane of Gram-negative bacteria. This destabilizes lipopolysaccharides or lipooligosaccharides, disrupting the bacterial cell envelope. However, resistance to polymyxin, both chromosomal and plasmid-mediated, is increasing and has been detected in various Gram-negative bacteria [79]. Several authors have studied the combination between polymyxin B and cannabinoids.

In the study by Abichabki et al. [30], CBD showed antibacterial activity against several Gram-negative bacteria, including multidrug-resistant strains (e.g., *K. pneumoniae*, *E. coli,* and *A. baumannii*), with concentrations below 4 μg/mL being effective when combined with polymyxin B [28]. In another study, Gildea et al. [42] showed that the growth of *S. typhimurium* was inhibited at very low doses of CBD–antibiotic co-therapy, specifically with 0.5 μg/mL ampicillin + 1 μg/mL CBD and 0.5 μg/mL polymyxin B + 1 μg/mL CBD. This synergistic antibacterial activity between polymyxin B and CBD was also shown by Hussein et al. [45]. Farha et al. [39] demonstrated that CBG, which was inactive against *E. coli* (with a concentration >128 μg/mL), exhibited significant potentiation when combined with a sublethal concentration of polymyxin B (1 μg/mL in the presence of 0.062 μg/mL polymyxin B). These studies suggest that the permeabilization of the outer membrane caused by polymyxin B is sufficient to allow the entry and antibacterial activity of cannabinoids (CBD and CBG).

Regarding the outcomes of co-therapy, Cham et al. [37] demonstrated additive effects of a semisynthetic phytocannabinoid, tetrahydrocannabidiol (THCBD, 4) with tetracycline, mupirocin, and penicillin G in *S. aureus*. Dihydrocannabidiol, (H_2_CBD) demonstrated synergistic or additive effects against *E. faecalis* and *Bacillus cereus* when combined with several antibiotics (tetracycline, gentamicin, ofloxacin, and chloramphenicol) [53]. In the work by Wassman et al. [57], CBD potentiated the effect of bacitracin against Gram-positive bacteria (*Staphylococcus* species, *Listeria monocytogenes*, and *E. faecalis*) but appeared ineffective against Gram-negative bacteria.

As cannabinoids are receiving significant research attention for their potential benefits in various applications, recent studies have observed their antimicrobial properties against the bacteria found in dental plaque. CBG showed antibacterial effects against *Streptococcus mutans*, the main aetiological agent of dental caries, inducing membrane hyperpolarization and preventing reduction in pH, which is normally caused by this microorganism and allows demineralization of enamel during the process of caries development [33]. Avraham et al. [34] concluded that the combination of triclosan and CBD demonstrated stronger antibacterial and anti-biofilm effects compared to each compound alone. Both compounds induced membrane hyperpolarization, reducing bacterial viability and adhesion. This combined treatment may be useful for preventing dental caries and oral inflammation [34]. Cannabinoid (CBD or CBG)-infused mouthwashes demonstrated similar bactericidal efficacy to chlorhexidine 0.2% [56].

### 4.2. Antiviral Activity of Cannabinoids—Recent Findings and Interpretation

Viruses are infectious agents that have the ability to invade the human body through a variety of routes, including respiratory, gastrointestinal, genitourinary, and skin routes. Once inside the body, these agents use the host’s cells to replicate their genetic material, i.e., DNA or RNA, depending on the type of virus. Although the immune system is usually effective in fighting off most viral infections, in some cases, the virus is so aggressive that another method must be used because the immune system cannot eradicate the infection on its own [80]. 

Just as there is currently a shortage of antibiotics to fight bacteria, there is also a severe shortage of antivirals. In the last four years, the world has been forced to face an emerging infectious disease, the Severe Acute Respiratory Syndrome Coronavirus 2 (SARS-CoV-2) pandemic, due to the lack of therapies capable of fighting this infection and the mortality observed, associated with the excessive production of pro-inflammatory cytokines such as interleukins, interferons, and tumor necrosis factor-α (TNF-α) [81]. Studies have therefore been carried out on the activation of the endocannabinoid system as a possible treatment for this infection since the effects of cannabinoids on the immune system have the potential to limit the abnormal functioning of this system when the body is infected, thus reducing the mortality caused by this virus [80].

Regarding the antiviral effect of CDB, most *in vitro* studies within this scoping review are related to its potential antiviral effect against SARS-CoV-2. In accordance with several studies, CBD exhibited the highest antiviral activity against a panel of SARS-CoV-2 variants by reducing viral entry by affecting virus spike protein-mediated membrane fusion [55,56,58,64]. Van Breemen et al. [70] also reported that cannabinoid acids were found to be allosteric as well as orthosteric ligands with micromolar affinity for the spike protein.

The CB_1_ receptor, which is extensively distributed throughout the central nervous system, plays a key role in influencing viral infections in neural, lung, and liver tissues when activated by cannabinoid agonists. [82]. In certain viral infections, the activation of CB_1_ receptors can trigger signaling pathways that lower cellular calcium ion levels, leading to a disruption in the release of calcium-dependent enzymes, nitric oxide production, nitric oxide synthase activity, and pro-inflammatory mediators. These compounds contribute negatively by enhancing the host’s response to the viral infection and facilitating viral replication. The activation of CB_2_ receptors on immune cells modifies the immune response and impacts viral infections. CB_2_ receptors’ immunomodulatory and anti-inflammatory effects can reduce immune activity, suppress inflammation, regulate cytokine production, and influence the migration of immune cells [81,83]. Δ^9^-THC is known to be a partial agonist for both endocannabinoid receptors, inducing psychotomimetic effects by activating the CB_1_ receptor while also influencing the immune system through its binding to CB_2_ receptors. In contrast, CBD plays a role in regulating immune responses, has minimal or no psychotomimetic effects, and functions as a CB_1_ receptor antagonist and a CB_2_ receptor agonist [84].

The anti-inflammatory effects of cannabinoids are mediated through various pathways, including the regulation of immune cell production, migration, and function (such as macrophages, monocytes, neutrophils, lymphocytes, dendritic cells, NK cells, fibroblasts, and endothelial cells). They also reduce the levels of pro-inflammatory cytokines (e.g., IL-1β, IL-2, IL-6, IL-8, IL-12, IL-17, IL-18, IFN-γ, TNF-α, MCP-1/CCL5, GM-CSF) and promote the production of anti-inflammatory cytokines (such as IL-4, IL-10, IL-11, TGF-β) [80].

Mahmud et al. [81] reviewed the pharmacological potential of cannabinoids on the SARS-CoV-2 virus and concluded that intranasal administration of CBD led to a decrease in the pro-inflammatory secretion of the cytokine IL-6, resulting in an improvement in symptoms associated with COVID-19. The authors also concluded that, in humans, oral administration of Δ^9^-THC and CBD significantly reduced TNF-α levels, with CBD identified as a PPARγ agonist. This action may contribute to its antiviral effects and help suppress the onset of the cytokine storm in COVID-19 infections. Additionally, CBD regulates fibroblast/myofibroblast activation and inhibits the development of pulmonary fibrosis, leading to improved lung function in patients recovering from the disease.

The SARS-CoV-2 virus has evolved new strategies to manipulate host signaling pathways, such as the interferon pathway, to enhance its replication. While this provides an advantage to the virus, it poses a significant disadvantage to the population [85]. Nguyen et al. [63] found that CBD can inhibit SARS-CoV-2 replication by inducing host endoplasmic reticulum stress, increasing reactive oxygen species accumulation, and stimulating antiviral interferon production. It also enhances interferon-stimulated gene expression, suggesting the involvement of the interferon pathway in its antiviral effects. In later stages of infection, CBD and Δ^9^-THC reduce virus-induced cytokine release and immune cell recruitment, helping to prevent a cytokine storm. CBD, as a CB_2_ agonist, inhibits the TLR4/NF-κB signaling pathways, which are key drivers of pro-inflammatory cytokine expression. Thus, CBD shows potential as an antiviral agent that can both prevent viral replication in the early stages and suppress the immune response in later stages. Additionally, the cannabinoids CBGA and CBDA have been found to bind strongly to the spike protein, blocking viral infection [63,84].

Regarding other viruses, Marquez et al. [62] found that CBD affects cellular membranes, inhibiting the replication of Zika virus (ZIKV) and other viruses. ZIKV, primarily transmitted by *Aedes aegypti* mosquitoes, causes neurological diseases like microcephaly and Guillain–Barré syndrome. Despite recent outbreaks, there are no vaccines or specific treatments for ZIKV. The study demonstrated that CBD inhibits a range of structurally diverse viruses, suggesting it has broad-spectrum antiviral properties, making it a potential alternative in emergency situations during viral outbreaks, such as the COVID-19 pandemic.

### 4.3. Antifungal Activity of Cannabinoids–Recent Findings and Interpretation

Fungal infections, both superficial and systemic, have increased due to the emergence of various immunological disorders. Resistance to antifungal drugs has become a growing concern, highlighting the urgency of finding new therapeutic alternatives. *Candida albicans* is a pathogenic dimorphic fungus that opportunistically causes various types of infections in humans. Although only a few studies on the antifungal effect of CBD are available, there is agreement between authors concerning CBD antifungal activity against *Candida* sp, especially *C. albicans*. CBD inhibits the growth and formation of *C. albicans* biofilm and induces disorganization of mature biofilm [47,72,73,74,75]. The causes are still unclear, but there appears to be an inhibitory effect on the development of hyphae, which is recognized as a key pathogenic mechanism of *C. albicans.* Studies by Feldman et al. [73] reported that CBD repressed the expression of *C. albicans* virulence-associated genes (lipases, phospholipases, and cell wall proteins); enhanced the production of ROS, reducing the antioxidant defense genes SOD and causing mitochondrial dysfunction; and reduced intracellular ATP levels and subsequent apoptosis of *C. albicans* cells. CBD inhibited *C. albicans* biofilm formation by upregulating the DPP3 gene (associated with the biosynthesis of farnesol that inhibits biofilm formation) and downregulating ergosterol biosynthesis-associated genes (ERG11 and ERG20) [73]. Bahraminia et al. [74] reported that the inhibition of *C. albicans* by CBD was achieved through a combination of apoptosis and necrosis pathways. Additionally, this study demonstrated a synergistic effect of CBD combined with amphotericin B, enhancing its ability to inhibit the growth of *C. albicans* [67]. However, further studies are needed to reach clearer conclusions that could allow the use of *C. sativa* in fungal infections, mainly for external use.

## 5. Conclusions and Future Opportunities

The unique chemical properties of cannabinoids, combined with their interactions with existing therapies, contribute to their antimicrobial effects against a wide range of microorganisms, including bacteria, fungi, and viruses.

The data collected support the conclusion that cannabinoids exert their effects through multiple pathways, including the disruption of microbial membranes, modulation of immune responses, and interference with microbial virulence factors. The use of cannabinoids as alternative therapeutic options has demonstrated their potential to overcome the limitations of conventional antibiotics, offering a potential new approach to combating drug-resistant microorganisms, potentially reducing dependence on traditional antimicrobial agents that have become less effective. It also appears that the use of combinations of cannabinoids with other conventional drugs can potentially lead to a synergistic effect with improved therapeutic capabilities.

The scientific evaluation of the medical benefits of *Cannabis* sp. and its derivatives is hindered by several factors, including societal stigma, misinformation propagated by proponents of alternative medicine, legal restrictions, and health risks associated with Δ^9^-THC, particularly in children and adolescents. Nevertheless, the findings presented here underscore the importance of further investigating cannabinoid-based therapies, including their potential synergistic effects with existing antimicrobial agents. Equally important is the potential of combining cannabis-derived compounds with nanomedicine. This cutting-edge approach holds significant promise for addressing bacterial, viral, and fungal infections. By integrating the unique properties of cannabinoids and nanomaterials, this interdisciplinary strategy provides a novel pathway to enhance therapeutic outcomes against antimicrobial resistance and treatment-resistant pathogens. Further investigation of this promising avenue is warranted in the future.

Also, regarding the antibiofilm activity of CBD, more studies should focus on whether the antibiofilm effect is effective and assess its toxicity using *in vivo* models.

The pharmacological profiles of individual cannabis components and their mixtures with antibiotics, including absorption, distribution, metabolism, mode of action, elimination, and toxicity, need to be clearly defined. Further *in vivo* studies and preclinical trials with large participant groups are necessary. However, cannabis-based antimicrobial agents must meet strict regulatory requirements regarding quality, safety, efficacy, and cost-effectiveness, in line with good laboratory, manufacturing, and clinical/application practices.

## Data Availability

Not applicable.

## Supplementary Materials

The following supporting information can be downloaded at: https://www.mdpi.com/article/10.3390/microorganisms13020325/s1, Table S1. Search strategies used in the scoping review.
