# Supplementary material for: Antimicrobial Potential of Cannabinoids: A Scoping Review of the Past 5 Years
Source: Microorganisms. 2025 Feb 2;13(2):325. doi: 10.3390/microorganisms13020325 (PMC11858408; doi:10.3390/microorganisms13020325)
Supplement: Supplementary file 1 [file microorganisms-13-00325-s001.zip › microorganisms-3309025-supplementary.pdf]

Supplementary Material

Table S1. Search strategies used in the scoping review.

Electronic Database	Search strategy
PubMed	("cannab*"[Title/Abstract] AND ("antimicrobial*"[Title/Abstract] OR "antibacterial*"[Title/Abstract] OR "antiviral*"[Title/Abstract] OR "antifungal*"[Title/Abstract] OR "Antibiofilm"[Title/Abstract])) NOT "review"[Publication Type]) AND (2020/1/1:2024/10/31[pdat])
ScienceDirect	Title, abstract, keywords: (cannabis OR cannabinoid) AND (antimicrobial OR antibacterial OR antiviral OR antifungal OR antibiofilm)
Scopus	TITLE-ABS-KEY (antimicrobial OR antibacterial OR antifungal OR antiviral OR antibiofilm) AND TITLE-ABS-KEY(cannab*) AND PUBYEAR > 2019 AND PUBYEAR < 2025 AND (LIMIT-TO (DOCTYPE,"ar")) AND (LIMIT-TO (LANGUAGE,"English"))
